# Surgical Outcome in Extratemporal Epilepsies Based on Multimodal Pre-Surgical Evaluation and Sequential Intraoperative Electrocorticography

**DOI:** 10.3390/bs11030030

**Published:** 2021-03-04

**Authors:** Lilia María Morales Chacón, Judith González González, Martha Ríos Castillo, Sheila Berrillo Batista, Karla Batista García-Ramo, Aisel Santos Santos, Nelson Quintanal Cordero, Marilyn Zaldívar Bermúdez, Randis Garbey Fernández, Bárbara Estupiñan Díaz, Zenaida Hernández Díaz, Juan E. Bender del Busto, Abel Sánchez Coroneux, Margarita M. Báez Martin, Lourdes Lorigados Pedre

**Affiliations:** International Center for Neurological Restoration, National Epilepsy Surgery Program, 25th Ave, No 15805, Havana PC 11300, Cuba; judith@neuro.ciren.cu (J.G.G.); martharios@neuro.ciren.cu (M.R.C.); sheylabb@infomed.sld.cu (S.B.B.); kbatista.gr@gmail.com (K.B.G.-R.); aisel.santos@gmail.com (A.S.S.); nquintanal@neuro.ciren.cu (N.Q.C.); marilyn@neuro.ciren.cu (M.Z.B.); randis0770@gmail.com (R.G.F.); baby@neuro.ciren.cu (B.E.D.); zmhernandez@neuro.ciren.cu (Z.H.D.); jebender@infomed.sld.cu (J.E.B.d.B.); abel@neuro.ciren.cu (A.S.C.); minou@neuro.ciren.cu (M.M.B.M.); lourdes.lorigados@infomed.sld.cu (L.L.P.)

**Keywords:** extratemporal epilepsy surgery, multimodal neuroimaging, intraoperative electrocorticography, seizure outcome

## Abstract

Objective: to present the postsurgical outcome of extratemporal epilepsy (ExTLE) patients submitted to preoperative multimodal evaluation and intraoperative sequential electrocorticography (ECoG). Subjects and methods: thirty-four pharmaco-resistant patients with lesional and non-lesional ExTLE underwent comprehensive pre-surgical evaluation including multimodal neuroimaging such as ictal and interictal perfusion single photon emission computed tomography (SPECT) scans, subtraction of ictal and interictal SPECT co-registered with magnetic resonance imaging (SISCOM) and electroencephalography (EEG) source imaging (ESI) of ictal epileptic activity. Surgical procedures were tailored by sequential intraoperative ECoG, and absolute spike frequency (ASF) was calculated in the pre- and post-resection ECoG. Postoperative clinical outcome assessment for each patient was carried out one year after surgery using Engel scores. Results: frontal and occipital resection were the most common surgical techniques applied. In addition, surgical resection encroaching upon eloquent cortex was accomplished in 41% of the ExTLE patients. Pre-surgical magnetic resonance imaging (MRI) did not indicate a distinct lesion in 47% of the cases. In the latter number of subjects, SISCOM and ESI of ictal epileptic activity made it possible to estimate the epileptogenic zone. After one- year follow up, 55.8% of the patients was categorized as Engel class I–II. In this study, there was no difference in the clinical outcome between lesional and non lesional ExTLE patients. About 43.7% of patients without lesion were also seizure- free, *p* = 0.15 (Fischer exact test). Patients with satisfactory seizure outcome showed lower absolute spike frequency in the pre-resection intraoperative ECoG than those with unsatisfactory seizure outcome, (Mann– Whitney U test, *p* = 0.005). Conclusions: this study has shown that multimodal pre-surgical evaluation based, particularly, on data from SISCOM and ESI alongside sequential intraoperative ECoG, allow seizure control to be achieved in patients with pharmacoresistant ExTLE epilepsy.

## 1. Introduction

Extratemporal epilepsy (ExTLE) embraces a variety of seizures which can arise from the cerebral cortex outside of the temporal lobe [1]. Thus, epilepsy surgery constitutes an effective treatment for carefully selected patients with pharmacoresistant epilepsies, even when the outcomes of surgical treatment in ExTLE are less satisfactory compared to temporal lobe epilepsy (TLE) [2,3]. On the other hand, patients with magnetic resonance–negative focal epilepsy show less favorable surgical outcomes compared to those in whom an magnetic resonance imaging (MRI) lesion guides the site of surgical intervention [3,4].

Surgical treatment of ExTLE is still challenging due to hitches in defining the epileptogenic zone (EZ). Nonetheless, current advances in noninvasive techniques such as epilepsy specific MRI and functional neuroimaging—single photon emission-computed tomography (SPECT) and positron emission tomography (PET)—have improved the diagnostic tools of ExTLE, facilitating surgical treatment [4,5,6]. Equally, intraoperative electrocorticography (ECoG) may provide significant information concerning electrographic activity, which modifies the resection extension [7].

Apart from the ambiguity regarding the choice of the most prospective candidates, surgical treatment of ExTLE still has difficulties in localizing and defining the extension of the EZ. This paper summarizes post-surgical assessment in both lesional and non lesional extratemporal epilepsies patients, submitted to a preoperative multimodal evaluation including neuroimaging; explicitly, subtraction of ictal and interictal SPECT co-registered with magnetic resonance imaging (SISCOM) and electroencephalography (EEG) source imaging (ESI) of ictal epileptic activity as well as sequential intraoperative ECoG during surgical resective and/or disconnective procedures. 

## 2. Subjects and Methods

Patients with pharmacoresistant epilepsy were referred from all regions of the country covering urban and rural areas. Eligibility criteria required individuals to be non-responsive to at least two appropriate antiepileptic drugs (AEDs) trials due to inefficacy or intolerance; hence, recurrently compromised by seizures [8].

Family and patient’s consent was received in all cases. Subjects submitted to ExTLE epilepsy surgery with one-year follow-up after surgical procedure were included in this communication whereas those with prior brain operation were left out. In addition, clinical outcome data were collected prospectively at the International Center for Neurological Restoration, Havana Cuba from 2016 until 2019.

Preoperative evaluation included: (a) prolonged video-electroencephalography (VEEG) monitoring with scalp electrodes and additional electrodes considering the epileptogenic zone presumed; (b) MRI scans with a 1.5 T or 3T scanner (Siemens Magnetom Symphony); (c) a comprehensive battery of neuropsychological tests (executive functions, attention and memory assessment, higher verbal and visual functions) and; (d) multimodal evoked potentials, somatosensory, visual and auditive [9,10,11]. Interictal and ictal brain single photon emission computed tomography with EEG co-registration was also carried out in patients with non-visible lesion in MRI. Additionally, ictal ESI, SISCOM and MRI post processing were performed in this patient group in accordance with our previously published protocol [10].

### 2.1. Video Electroencephalography (EEG)-Based Diagnostics 

Patients underwent video-EEG monitoring for 8.7 ± 2.7-day. The distribution of interictal epileptiform discharges (IEDs) during prolonged video-EEG monitoring was assessed by (LM) through the analysis of 15-min-interictal EEG samples every one hour. The data recorded in relation to seizures were identified by button presses or by seizure or spike detection programs.

Furthermore, interictal epileptiform activity and ictal onset pattern were categorized as: (1) regional involving one lobe, and ipsilateral contiguous or, (2) non-regional. Ictal and interictal video-EEG were examined by a highly qualified epileptologist involved in this study (LM). One year following surgery, extracraneal prolonged EEG was also recorded (data not reported).

### 2.2. Pre-Surgical Neuroimaging-Based Diagnostics

Pre-surgical 1.5 (n = 13) or 3T (n = 21) MRI scans of the patients integrating T1-weighted images with and without gadolinium-DTPA, T2-weighted images, fluid-attenuated inversion recovery images, and magnetization-prepared rapid gradient echo sequences were reviewed by a versed neuroradiologist (ZH). 

MRI findings were classified as (1) MR visible/MR non-visible; (2) tumor, cortical development malformation, vascular lesions, among others; (3) eloquent cortex/non-eloquent adjacent to or overlapping with eloquent areas—the primary motor cortex or Broca’s area, sensorial, language-based on anatomic landmarks; and (4) laterality—dominant hemisphere/non-dominant. Moreover, eloquent cortical areas were designated according to Chang et al.’s classification, which comprised the rolandic cortex (pre- and postcentral gyrus), the supplementary motor area (SMA), insula, and primary visual cortex as well as Broca and Wernicke’s areas [12]. 

### 2.3. Single Photon Emission-Computed Tomography (SPECT) Co-Registered with Magnetic Resonance Imaging (MRI) (SISCOM) 

Brain perfusion SPECT was carried out in patients with non lesional extratemporal epilepsies. SPECT image acquisition was performed using a double-headed gamma camera (SMV DST-XLi, Buc Cedex, France) equipped with a fan-beam collimator. For co-registration with the MRI scan, the cerebral surface of the MRI volume was segmented from the extracerebral structures. Subsequently, the cerebral surface of the binary ictal SPECT was matched to the cerebral surface of the binary MRI. The resulting transformation matrix was then applied to the subtraction SPECT to co-register it to the cerebral surface of the MRI. Furthermore, each patient underwent two studies (Ictal and Inter-ictal) of brain perfusion SPECT using 99mTc-ethylene-cysteine dimer (ECD). In both studies, the subject remained monitored by EEG during the administration of the 99m Tc ECD. For ictal SPECT, the radiotracer was injected when the EEG seizures onset was identified. For inter-ictal SPECT, the dose of the radiotracer was administered with at least a 24 h- seizure-free period.

### 2.4. Ictal Electroencephalography Source Imaging (ESI)

The cortical generators of EEG measurements can be estimated by solving an inverse imaging problem where the unknown sources are distributed on the individual’s cortex. The methodology followed in this study for the estimation of the inverse solution of ictal EEG has previously been published [10].

### 2.5. Surgical Procedures and Histopathology

The extension of resection in lesional and non lesional patients was adjusted bearing in mind pre-surgical evaluation, and tailored by sequential pre- and post-resection ECoG. Data acquisition was performed with a Medicid-5 digital Electroencephalographic system (Neuronic SA, Cuba) with 32 channels, 256 Hz sampling rate and a 16 bit analogue-to-digital converter. Data were band-pass filtered between 0.53 and 70 Hz. ECoG analyses were performed by two board-certified electroencephalographers. Additionally, Ad-Tech subdural electrodes (grid and strips) were used. Then, quantification of discharges in each register-electrode was determined by using the Neuronic automatic spike detection system. Two clinical neurophysiology specialists visually reviewed these results for artifacts (LM, SB). All spike activity regarded as artifactual was excluded. Lastly, the absolute spike frequency (ASF, spike/min) was calculated in the pre- and post-resection ECoG. 

Accurate identification of lesion localization relative to eloquent cortex was derived from intraoperative ECoG using cortical mapping with evoked potentials and electrical stimulation. Moreover, subtotal resection was intentionally performed when the lesion overlapped with eloquent cortex.

Histopathological findings comprised four chief groups: cortical development malformations, neoplasms, vascular lesions, and other non-specific histopathological abnormalities. In cases of mycroscopic diagnosis, and focal cortical dysplasia (FCD) classification, the system proposed by the International League Against epilepsy was used [13]. For histopathological diagnosis of central nervous system tumors, the World Health Organization classification was employed [14]. Neoplasms were categorized as glial tumors (astrocytomas, oligoastrocytomas, and oligodendrogliomas) and neuroepithelial tumors (gangliogliomas and dysembryoplastic neuroepithelial tumors, DNT). On the other hand, non-specific histopathological abnormalities included gliosis, scars, among others were also tabulated.

### 2.6. Seizure Outcomes

Seizure outcome assessment was based on the system proposed by Engel [Engel class I, free of disabling seizures; class IA, seizure-free; class II, rare seizures (less than three seizures per year); class III, worthwhile improvement (reduction in seizures of 80% or more); class IV, no benefit] [15]. In general, patients classified as Engel class I or II were categorized as satisfactory seizure outcome while those included in Engel class III or IV were labelled as unsatisfactory. Subjects were routinely evaluated 12 months following surgery. 

### 2.7. Statistics Analysis

Indicators were summarized with descriptive statistics for each variable comprising mean, median, and standard deviations for continuous variables, and frequencies for categorical ones. STATISTICA (data analysis software system), version 8.0, www.statsoft.com.Tulsa, USA). Finally, Mann Whitney and Wilcoxon tests were applied for independent and dependent samples respectively. Exact *p* values generated for small and moderate samples were taken for significance evaluation, then statistical significance was set at *p* < 0.05.

### 2.8. Ethical Considerations

The procedures performed followed the 1975 Declaration of Helsinki for human research. Correspondingly, this study was approved by the scientific and ethics committee of the International Center for Neurological Restoration (CIREN37/2012).

## 3. Results

### 3.1. Preoperative Evaluation

Thirty-four patients (28 males) were included in this study (Table 1). Mean age at surgery was 24.38 years (standard deviation 8.8, range 8–47) with average epilepsy duration of 16.79 years (standard deviation 9.5 ranged 3–42). Mean age at seizure onset was 7.6 ± 5.7 (ranged 5 months to 21 years) Table 1, and pre-surgical seizure frequency was 20/months or more in 76.4% (26). Also, risk factors were considered in 27 of the patients. 

All participants who matched the selection criteria were taking 2–4 antiepileptic drugs (AEDs). Lamotrigine, carbamazepine, clonazepan, valproic acid, clobazan and levetiracetan were the most frequent prescriptions, and the mean number of antiepileptic drugs at surgery time was 2, 87 ± 0.83. 

#### Multimodal Pre-Operative Assessment

During extracranial video-EEG monitoring, a mean of 20.6 ±15.9 seizures per patient was recorded with a mean video-EEG monitoring efficiency equal 0.77. Data with reference to awake and sleep seizures day-to-day were 1.55 and 0.9, respectively.

Furthermore, regional interictal EEG pattern was recorded in 53.8% (18) of the patients while 74% (25) exhibited non-lateralized or bilateral interictal epileptiform discharges (IED). In contrast, ictal EEG pattern was lateralized in 71.4% (24) and regional in 82.3% (28) of the subjects. Non-aware focal seizures were the most frequent seizure type, 38.2% (13/34), which then evolved to bilateral tonic clonic seizures. On the other hand, aware focal seizures changed to non-aware and bilateral tonic clonic seizures, which was noticed in 26.4% (9/34). Non-epileptic seizures were also reported in two of the patients along with epileptic seizures. 

Although the attention in this work is not focused on the evaluation of the neuropsychological functioning in these patients, it is important to note that no significant differences were found in the executive function scales between pre- and post-operative evaluations. *p* = 0.32 Wilcoxon matched pairs test. Furthermore, impairment in the domain of phonological fluency was evidenced in the pre-operative evaluation in 84% of the patients, and 80%. *p* = 0.32 in the post-surgery stage.

**Figure 1:**
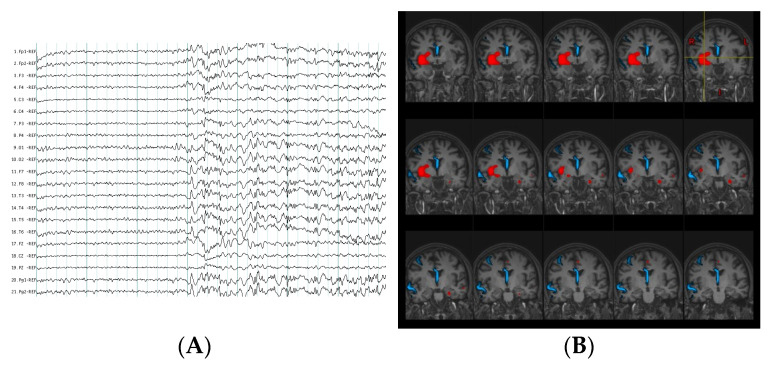
Multimodal evaluation in non-lesional extratemporal epilepsy patient. (**A**) Ictal scalp electroencephalography (EEG) pattern at seizure onset during habitual non aware focal motor seizures which evolved to bilateral tonic clonic seizures. Visual EEG localization did not show a clear lateralized and localized seizure onset zone. (**B**) In red, computer- aided subtraction ictal single photon emission-computed tomography (SPECT) co-registered to MRI (SISCOM) of the patient indicated localized areas of hyperperfusion (insula, inferior opercular frontal, putamen, amygdala, and anterior cingulum of the right hemisphere). In blue, estimation of ictal EEG source imaging (ESI) discharges at seizure onset also demonstrated a localize ictal source in this patient (right middle frontal gyrus, right superior temporal and middle line).

Magnetic resonance imaging did not indicate a distinct lesion in 16 patients (47%), 13 of whom were submitted to a methodology combining non-invasive functional modalities, subtraction of ictal and interictal SPECT co-registered with magnetic resonance imaging (SISCOM) and EEG source imaging (ESI) of ictal epileptic activity to estimate the location of the EZ. The findings of both methodologies showed high relation to the resection zone in Engel I–II subjects Figure 1.

### 3.2. Epilepsy Surgery Procedures and Surgical Outcome 

#### 3.2.1. Surgical Techniques were Classified as Resective, Disconnective and Combined

Adjusted frontal lobectomy 21 (61.7%), as well as occipital and parietal were the most common resection procedures. About 65.5% (19/29) of the resective surgeries was done in non-dominant hemispheres whereas 14 (41.17%) of the ExTLE patients undertook surgical resection encroaching upon the eloquent cortex. Multiple subpial transection was undertaken additionally to resection in eloquent areas in six of the subjects (three in frontal, and three in pericentral cortex). Both focal resection and anterior callosotomy were carried out in six of the cases. The four disconnective procedures performed included two frontal and two occipital Table 1.

#### 3.2.2. Surgical Outcome and Intraoperative ECoG 

**Figure 2:**
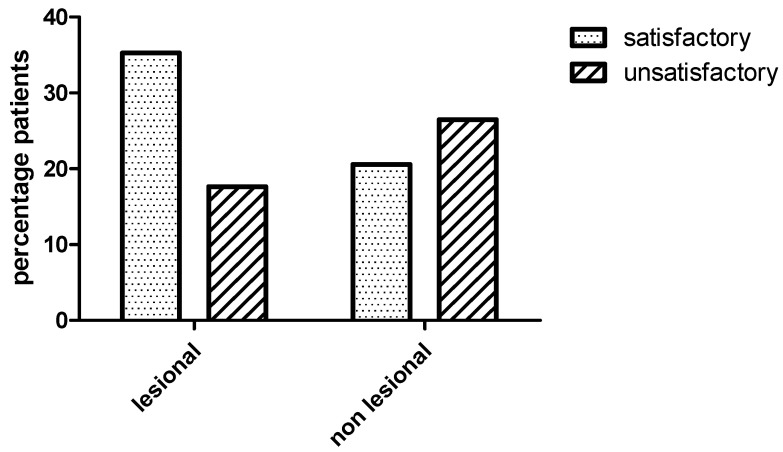
Bar graph showing clinical outcome between lesional and non lesional extraemporal epilepsies patients. There was no difference in the clinical outcome between lesional and non lesional patients (Fischer exact test, *p* = 0.15). Satisfactory (Engel class I–II) and unsatisfactory (Engel class III–IV) outcome one year after surgery.

After one-year follow up, 19/34 (55.8%) of the patients had a satisfactory seizure outcome (Engel class I–II). In this arm, the highest frequency was occupied by cases classified within class I, 16/19 (84, 2%). Engel scores follow-up for this group were as follows: 9 class IA, 5 IB, 2 IC and 3 II. On the other hand, Engel scores follow-up for unsatisfactory seizure outcome (Engel class III–IV) patients was described as 7 class III and 8 class IV. In the current study, there was no difference in the clinical outcome between lesional and non lesional ExTLE patients, *p* = 0.15, Fischer exact test Figure 2. Of the 19 patients who were seizure-free, seven had no macroscopic lesion in MRI. 

One of the two patients reported who had non-epileptic seizures in addition to epileptic ones was seizure-free for both epileptic and non-epileptic seizures whereas the other was classified as Engel IVA class, screening a decrease in the frequency of non-epileptic seizures evaluated by video-EEG. 

**Figure 3:**
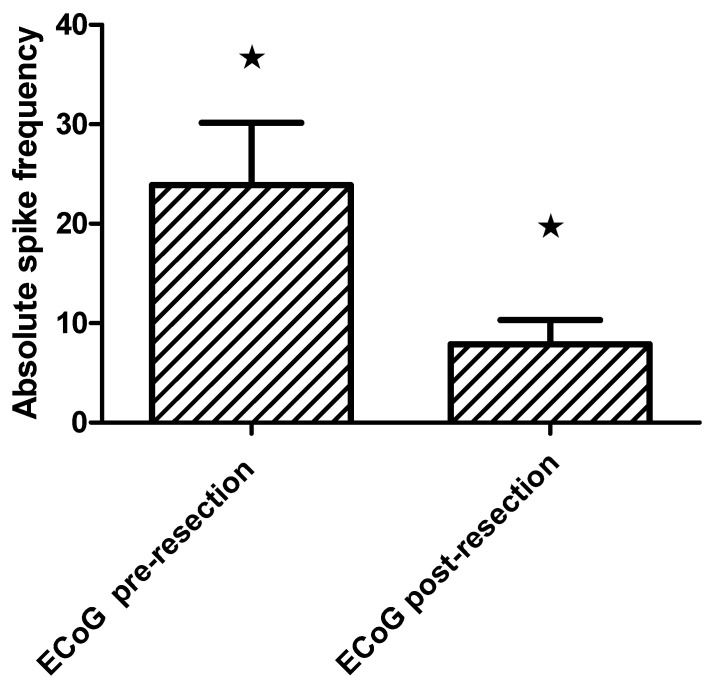
Bar grah showing absolute spike frequency on the pre and post-resection intraoperative electrocorticography (mean and standard error SE) in extratemporal epilepsies patients (Wilcoxon matched pairs test, * *p* = 0.002).

**Figure 4:**
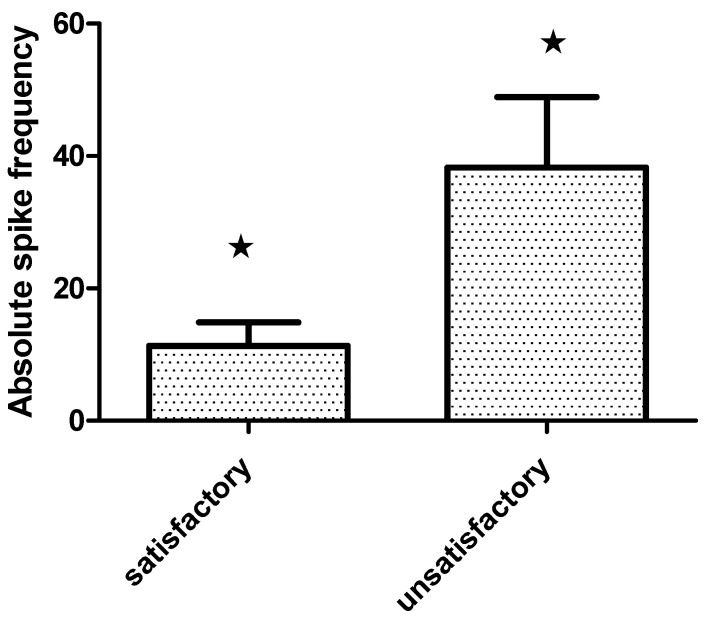
Bar grah showing absolute spike frequency on the pre-resection intraoperative Electrocorticography·(ECoG) (mean and standard error SE) in extratemporal epilepsies patients with satisladory·(Engel class I–II) and unsatisactory (Engel class III–IV) outcome one year after surgery (Mann Whitney U test, * *p* = 0.005).

All patients were submitted to pre-resection and sequential post-resection ECoG. Repetitive interictal spikes and other specific patterns were seen in 79.4% (27). The absolute spike frequency diminished significantly in the last post-resection ECoG (Wilcoxon matched pairs test, *p* = 0.002) Figure 3. Furthermore, patients with satisfactory seizure outcome showed lower absolute spike frequency in the pre-resection ECoG (11.3 ± 3.6/min) than those with seizure recurrence (38.3 ± 10.6/min), Mann–Whitney U test, *p* = 0.005 Figure 4. 

#### 3.2.3. Histopathological Findings 

As can be seen from Table 1, malformations of cortical development accounted for 21/28 (75%) of all histopathological findings accompanied by neoplasms. There was a similar proportion of patients with FCD type I 10/21 (47.6%), and Type II 11/21(52.3%), *p* = 0.74. In addition, neoplasms astrocytoma and ganglioglioma were observed in three patients. 

#### 3.2.4. Operative Complications 

Permanent neurological morbidity was detected in three of the patients (8.8%), described as paresis, dysphasia, and sightlessness. As shown in Table 1, there was no mortality in our cohort. On the other hand, one patient classified as Engel Class I died from cardiovascular disease 15 months post-surgery.

### 3.3. Discussion

The most important clinically relevant finding was that 55.8% of the patients with extratemporal epilepsy had a satisfactory seizure outcome (Engel class I–II), of whom 84, 2% were in Engel class I. The clinical outcome was similar in lesional and non lesional. ExTLE patients. In the second group, a relatively high incidence of FCD type I was found. The findings of this study suggest that multimodal evaluation combined with sequential intraoperative ECoG can facilitate satisfactory seizures outcome.

There is evidence that patients with a lesion in the MRI show the best seizure outcome after surgical procedures in temporal and extratemporal epilepsies [16,17,18,19,20,21,22,23,24,25,26]. However, it is important to note that, in our series, almost half of the patients who had non-lesional epilepsy were submitted to surgery without invasive EEG. It is interesting to signal that most of our patients exhibited non-aware focal seizures which then evolved to bilateral tonic clonic. During seizures, EEG pattern was predominantly regional and lateralized. However, interictal EEG pattern was regional and non-lateralized. 

It is a widely held view that surface electroencephalography is an essential method for the diagnosis, characterization and localization of extratemporal neocortical epilepsies; however, it has low sensibility compared to identical application for temporal epilepsies. In addition, interictal epileptiform discharges (IED) occur in 60% to 80% of frontal lobe epilepsy, and are considered to be of less localizing value than in TLE as they can be bilateral, multilobar or even generalized. Evidence of focal seizure onset can also be derived from regional EEG slowing or spikes [11,20,21].

There is strong evidence that for occipital, frontal, and parietal lobe epilepsies, the evaluation and localization of epileptic discharges by surface EEG are very challenging. This determines the difficulties not only to localize but also to define a false lateralization. For occipital lobe epilepsies, the difficulty of lateralization is added to a diffusion of discharges, in many cases, to ipsilateral temporal lobe with clinical manifestations similar to those of temporal lobe [17]. Notably, intracranial EEG is often used to localize the area responsible for seizure, but as this is an invasive technique, it cannot sample the activity from the whole brain.

A multimodal evaluation, specifically the use of SISCOM and ESI, developed by our group enabled satisfactory outcomes without intracranial EEG. Brain perfusion, ictal and interictal SPECT, along with SISCOM offered a high criterion of veracity in ictal onset detection represented by an increase in cerebral blood perfusion [10]. Previous studies have reported a sensitivity of interictal SPECT of 44%, and ictal over 97% in temporal lobe epilepsy, contrary to a sensitivity of 66% in ictal and 40% interictal in extra-temporal epilepsy [5,6,10].

On the other hand, ESI allowed us to infer the configuration of neuronal sources responsible for ictal activity. A further 13/16 (81.25%) non-lesional epilepsy patients went through multimodal neuroimaging evaluation with SISCOM and ESI. The findings of both methodologies showed a high relation to the resection zone in satisfactory seizure outcome subjects. Recent progress in neurophysiological and neuroimaging techniques has not only significantly improved non-invasive pre-surgical evaluation, but also opened the choice of epilepsy surgery to patients not previously considered surgical candidates [5,6,10,11]. 

In this study there was no difference in the clinical outcome between non-lesional and lesional ExTLE patients. In the latter group, FCD was the most common histopathological finding, with similar proportion between FCD Type I and Type II. FCD has been identified as a major cause of pharmacoresistant extratemporal resections, especially in children and adolescents [27,28,29], with a seizure-free rate after resection between 52% and 68.9% [30,31,32]. 

In accordance with previous studies, we found a relatively high incidence of FCD type I among operated patients with normal MRI [33,34,35]. In this framework, some authors have pointed out that even the invisible underlying pathology, explicitly FCD, may represent a favorable prognostic indicator in case of complete removal of the EZ when compared with all other etiologies [36,37,38].

In a recent extratemporal series, FCD accounted for 46.5% of all histopathological findings together with tumors, gliosis, and cavernomas [3]. Similarly, astrocytomes and gangliogliomas were the tumors identified in our patients; being the latter of the group of long-term epilepsy-related tumors. With respect to histopathology, more favorable seizure outcomes have been described in patients with cavernomas and glioneuronal tumors (gangliogliomas and DNTs with 89% and 85% seizure-free (Engel I) patients, respectively. Consistent with other research, 2/3 (66.6%) of our patients with tumor conditions remained seizure-free. 

Even with the aforementioned neuroimaging and histopathological profile, our seizure outcome matches those observed in earlier studies. One year after surgery, 55.8% of the extratemporal epilepsies patients with lesional and non-lesional epilepsy were categorized as satisfactory seizure outcome (Engel I–II class). Likewise, the surgical outcome in our cohort is in agreement with a large case series of surgery for extratemporal lobe epilepsies reported, in which 49% of the patients were Engel IA at an average of 54 months post-operatively [3]. In Delev D’s report, Engel I outcome after frontal and parietal resections was 65% and 71%, correspondingly, while other studies informed Engel I outcome ranging from 45.1% to 57.5% [16,17,18]. The results of this study are also in line with a meta-analysis described by Tellez-Zenteno et al., and slightly better compared to other series [19]. Some specialists from developing countries involved in temporal and extratemporal epilepsy surgery have reported Engel class I outcome in about 60% at 12 months’ follow-up [20,21,22].

Most procedures carried out for extratemporal epilepsies in this study are frontal resections [26], accounting for 61.7% of our cluster, followed by occipital, parietal and pericentral resection, or combined with disconnection techniques. This finding is in agreement with findings in Delev D’s series which described 48% of frontal lobe operations, and 24% parietal, occipital, and insular resections [3]. They also reported that the most positive epileptological outcomes were achieved in individuals with frontal and parietal resections (Engel I 65.0% and 71.4%, respectively), in contrast to insular resections, revealing less auspicious results (Engel I 52.2%).

Outstandingly, such comparisons are limited by both referral patterns and selection criteria, which are likely to fluctuate from different centers in Latin American countries. In order to homogenize these criteria, our cases were discussed in an epilepsy surgery conference including a multidisciplinary team of the epilepsy surgery program.

It has commonly been assumed that the success of epilepsy surgery depends upon accurate localization and complete resection of the epileptogenic tissue, which are both aided by intraoperative ECoG. Moreover, the presence of persistent spikes on post-resection ECoG has been a significant statistical association with poor seizure freedom post-surgery [39]. It is also recognized that intraoperative ECoG is a valuable adjunctive test in epilepsy surgery to accomplish ideal seizure freedom in cases of mesial temporal sclerosis plus focal cortical dysplasia and tumors. In the current study, a significant difference was observed between pre- and post-resection absolute spike frequency (ASF) during sequential intraoperative ECoG. 

In terms of complications, the rate is higher in extratemporal location compared to temporal resections with a reported perioperative mortality of 1.2% in extratemporal resections [40]. Appreciably, permanent morbidity of extratemporal procedures varies in different series between 3% and 43%. The most frequent harms include visual field defects, hemiparesis, aphasia, as well as cranial nerve palsies. There are other reports in which the neurological complications of resective surgery led to a temporary morbidity of 10.9% and a permanent morbidity of 4.7% [41,42,43]. Consistent with those of Delev D’s series, in this study, there was no mortality, and permanent morbidities were observed in three of the cases (8.8%), regardless surgical procedures [3]. This number corroborates those of the previous studies that have reported a permanent morbidity between 10% and 15% [3,23,24,44]. 

A limitation of this study is the relatively small number of patients that precluded the extraction of valuable information about potential prognostic factors in seizure recurrence. On the other hand, further research should be undertaken to evaluate the predictive value of the multimodal evaluation in ExTLE epilepsy surgeries. Nonetheless, the results of the current study support the possibility of conducting epilepsy surgery as an effective treatment for carefully selected patients with pharmaco-resistant extratemporal epilepsy. This study indicates that multimodal pre-surgical evaluation based on data resulting from video-EEG neuroimaging, specifically SISCOM and ESI in addition to sequential intraoperative ECoG, allow seizure control to be achieved in patients with pharmacoresistant extratemporal epilepsy.

## Figures and Tables

**Table 1:** Demographic, clinical and surgery profile.

Age at Surgery (years)	Seizure Onset (years)	Epilepsy Duration (years)	Sex	Epilepsy Type	Epilepsy Surgery Type	Histopathological Findings	Postperative Complications	Post-Surgery Outcome
25	9	16	female	LFE	L parietal lesionectomy	Tumor		Ia satisfactory
21	2	19	male	LFE/NESz	R frontal lobectomy	FCD IIa		IVa unsatisfactory
35	20	15	male	LFE	R frontal lesionectomy	Cavernous angioma		Ia satisfactory
47	5	42	female	LFE	L occipital lobectomy	Tumor	wound infection (T)	IIIa unsatisfactory
22	4	18	male	LFE	R frontal lesionectomy	FCD IIb		Ia satisfactory
20	3	17	male	NLFE	R frontal resection	Descriptive	Meningitis deep vein thrombosis (T)	IIIa unsatisfactory
44	6	38	male	LFE	L occipital lesionectomy	Meningio angiomatosys	sensitivy dysphasia (T)	Ia satisfactory
24	5	19	male	N LFE	R orbitofrontal lesionectomy	FCD I	sightlessness (P)	IV a unsatisfactory
27	18	9	male	LFE	R pericentral lesionectomy plus MST	FCD IIb	L monoparesis (T)	Ib satisfactory
21	8	13	male	N LFE	R orbitofrontal resection	FCD I		IV a unsatisfactory
17	14	3	female	LFE	R peri central resection	FCD IIb	cranial nerve palsies (T)	IV a unsatisfactory
26	3	23	male	LFE	R frontal resection plus MST	FCD IIb		IV b unsatisfactory
16	4	12	male	Lennox Gastaut Syndrome plus focal lesion	anterior callosotomy plus L frontal resection	FCD I		IVb unsatisfactory
38	8	30	male	LFE	R premotor frontal resection plus MST	Non useful tissue		IIa satisfactory
22	9	13	female	LFE/NESz	R parietotemporal lesionectomy	Tumor		Ia satisfactory
29	14	15	male	LFE	R frontal lesionectomy plus disconnection	FCD IIb	cerebrospinal fluid leak (T)	Ic satisfactory
22	5	17	male	NLFE	R midlle frontal gyrus topectomy plus MST	FCD I		IV a unsatisfactory
29	11	18	male	NLFE	R frontal resection	FCD 1c		Ib satisfactory
24	0	24	male	NLFE	R frontal lobectomy	Descriptive		Ib satisfactory
24	15	9	male	NLFE	R frontal Resection plus anterior callosotomy	FCD IIa		IIIa unsatisfactory
23	22	1	female	NLFE	L frontal resection plus anterior callosotomy	FCD IIa		IIb satisfactory
32	25	7	male	LFE	R occipital lobectomy and posterior temporal topectomy	FCD IIb	visual field defects (P)	Ia satisfactory
29	26	3	male	LFE	L frontal lesionectomy	FCD IIa		Ic satisfactory
32	11	21	male	LFE	L frontal topectomy	FCD Ia	Hemiparesis (P)	IVc unsatisfactory
37	31	6	male	N LFE	R superior frontal gyrus resection and midlle gyrus topectomy plus callosotomy	FCD Ia	disconnection syndrome (T)	Ia satisfactory
19	19	0	male	Lennox Gastaut Syndrome	anterior callosotomy	No tissue	disconnection syndrome (T)	IIIa unsatisfactory
21	3	18	male	LFE	L superior frontal gyrus corticectomy and midlle gyrus topectomy	FCD Ic		Ia satisfactory
18	10	8	male	NLFE	L parietal topectomy and posterior disconnection	FCD Ia		Ib satisfactory
18	15	3	male	NLFE	L frontal gyrus corticectomy plus callosotomy	descriptive	disconnection syndrome (T)	IIIa unsatisfactory
14	6	8	male	LFE	L frontal lesionectomy plus callosotomy	descriptive	epidural hematoma (T)	Ia satisfactory
11	10	1	male	Lennox Gastaut Syndrome plus focal lesion	R occipital disconnection	polymicrogyria		IIIa unsatisfactory
17	14	3	female	NLFE	L frontal resection plus MST	FCD Ia		IIa satisfactory
15	4	11	male	Lennox Gastaut Syndrome plus focal dysfunction	L frontal resection plus anterior callosotomy plus disconnection	descriptive	hemiparesis (T)	IIIa unsatisfactory
8	5	3	male	NLFE	R frontal resection plus MST	FCD I		Ib satisfactory

NESz: non epileptic seizures; NLFE: non lesional focal epilepsy; LFE: lesional focal epilepsy; FCD: focal cortical dysplasia; R: right; L: left; MST: multiple subpial transection; T: temporary; P: permanent; satisfactory: Engel Class I or II; unsatisfactory: Engel Class III or IV.
